# Tetra-μ-acetato-κ^8^ *O*:*O*′-bis{[(*E*)-2-styrylpyrazine-κ*N* ^3^]copper(II)}

**DOI:** 10.1107/S1600536810037487

**Published:** 2010-09-30

**Authors:** Jian-Guo Zhou, Mei Ming

**Affiliations:** a Department of Materials Science and Engineering, Tianjin Institute of Urban Construction, Tianjin 300384, People’s Republic of China; b Department of Basic Science, Tianjin Agricultural University, Tianjin 300384, People’s Republic of China

## Abstract

In the binuclear title compound, [Cu_2_(CH_3_COO)_4_(C_12_H_10_N_2_)_2_], the copper(II) ions are coordinated by four O atoms from two pairs of bridging acetate ligands and one N atom from a (*E*)-2-styryl­pyrazine ligand in a distorted tetra­hedral geometry. The structure displays no hydrogen bonding or π–π stacking inter­actions between the discrete binuclear entities.

## Related literature

For heterocyclic ligands as building tectons of the supra­molecular lattice in inorganic-organic coordination chemistry, see: Batten (2001 ▶); Kitagawa & Matsuda (2007 ▶); Moulton & Zaworotko (2001 ▶).
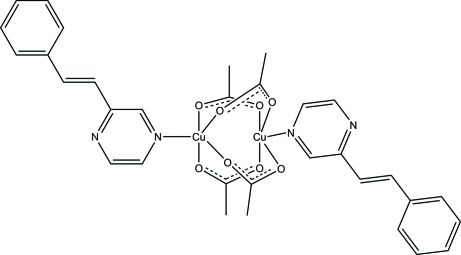


## Experimental

### 

#### Crystal data

[Cu_2_(C_2_H_3_O_2_)_4_(C_12_H_10_N_2_)_2_]
*M* *_r_* = 727.70
Triclinic, 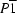

*a* = 10.519 (4) Å
*b* = 10.755 (4) Å
*c* = 15.924 (6) Å
α = 80.829 (6)°
β = 71.321 (6)°
γ = 74.300 (6)°
*V* = 1637.7 (10) Å^3^
*Z* = 2
Mo *K*α radiation
μ = 1.35 mm^−1^
*T* = 293 K
0.24 × 0.20 × 0.16 mm

#### Data collection

Bruker APEXII CCD area-detector diffractometer
Absorption correction: multi-scan (*SADABS*; Bruker, 2001 ▶) *T*_min_ = 0.750, *T*_max_ = 1.000
9491 measured reflections
6662 independent reflections
3702 reflections with *I* > 2σ(*I*)
*R*_int_ = 0.031

#### Refinement

*R*[*F* ^2^ > 2σ(*F* ^2^)] = 0.050
*wR*(*F* ^2^) = 0.109
*S* = 0.99
6662 reflections
419 parameters
H-atom parameters constrained
Δρ_max_ = 0.42 e Å^−3^
Δρ_min_ = −0.46 e Å^−3^

### 

Data collection: *APEX2* (Bruker, 2003 ▶); cell refinement: *SAINT* (Bruker, 2001 ▶); data reduction: *SAINT*; program(s) used to solve structure: *SHELXS97* (Sheldrick, 2008 ▶); program(s) used to refine structure: *SHELXL97* (Sheldrick, 2008 ▶); molecular graphics: *DIAMOND* (Brandenburg, 2005 ▶); software used to prepare material for publication: *SHELXTL* (Sheldrick, 2008 ▶).

## Supplementary Material

Crystal structure: contains datablocks I, global. DOI: 10.1107/S1600536810037487/jh2199sup1.cif

Structure factors: contains datablocks I. DOI: 10.1107/S1600536810037487/jh2199Isup2.hkl

Additional supplementary materials:  crystallographic information; 3D view; checkCIF report

## Figures and Tables

**Fig. 1.**
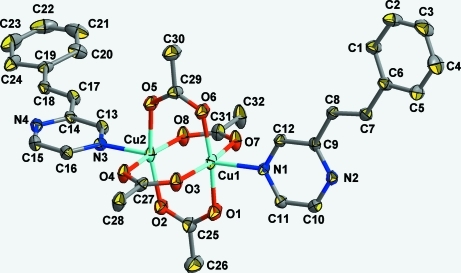
Representation of this complex with atomic labels of asymmetric unit and coordination sphere, shown with 30% probability displacement ellipsoids.

## supplementary crystallographic information

### Comment

Heterocyclic derivative ligands, as the excellent building tectons of supramolecular lattice, are very popular in the inorganic-organic coordination chemistry (Batten (2001); Kitagawa *et al.* (2007); Moulton *et al.* (2001)).

In this paper, (*E*)-2-styrylpyrazine was employed as a terminal ligand to assembly with Cu(OAc)_2_ to afford a binuclear complex, in which the Cu(II) displays a tetrahedral coordination geometry, and coordinated by four oxygen atoms from two pairs of acetates and one nitrogen donor from one (*E*)-2-styrylpyrazine ligand (see figure 1). The dimeric cage can be properly described as the paddle-wheel unit.

Further investigation on its supramolecular interaction reveals that no secondary contact such as hydrogen bonding and pi···pi stacking interaction is observed between these diecrete units.

### Experimental

A water (8 ml) solution containing Cu(OAc)_2_ (18.1 mg, 0.1 mmol) and (*E*)-2-styrylpyrazine (18.2 mg, 0.1 mmol) was heated to 100 \%C for 24 h and subsequently cooled to room temperature at a rate of 1 \%C/h. Blue block shape crystals were obtained.

### Refinement

All H atoms were initially located in a difference Fourier map. The C—H atoms were then constrained to an ideal geometry, with C—H distanceof 0.93 \%A, and *U*_iso_(H) = 1.2*U*_eq_(C).

### Crystal data

**Table:**

[Cu_2_(C_2_H_3_O_2_)_4_(C_12_H_10_N_2_)_2_]	*Z* = 2
*M**_r_* = 727.70	*F*(000) = 748
Triclinic, *P*1	*D*_x_ = 1.476 Mg m^−^^3^
*a* = 10.519 (4) Å	Mo *K*α radiation, λ = 0.71073 Å
*b* = 10.755 (4) Å	Cell parameters from 715 reflections
*c* = 15.924 (6) Å	θ = 2.5–23.8°
α = 80.829 (6)°	µ = 1.35 mm^−^^1^
β = 71.321 (6)°	*T* = 293 K
γ = 74.300 (6)°	Block, blue
*V* = 1637.7 (10) Å^3^	0.24 × 0.20 × 0.16 mm

### Data collection

**Table:**

Bruker APEXII CCD area-detector diffractometer	6662 independent reflections
Radiation source: fine-focus sealed tube	3702 reflections with *I* > 2σ(*I*)
graphite	*R*_int_ = 0.031
phi and ω scans	θ_max_ = 26.5°, θ_min_ = 1.4°
Absorption correction: multi-scan (*SADABS*; Bruker, 2001)	*h* = −7→13
*T*_min_ = 0.750, *T*_max_ = 1.000	*k* = −12→13
9491 measured reflections	*l* = −19→19

### Refinement

**Table:**

Refinement on *F*^2^	Primary atom site location: structure-invariant direct methods
Least-squares matrix: full	Secondary atom site location: difference Fourier map
*R*[*F*^2^ > 2σ(*F*^2^)] = 0.050	Hydrogen site location: inferred from neighbouring sites
*wR*(*F*^2^) = 0.109	H-atom parameters constrained
*S* = 0.99	*w* = 1/[σ^2^(*F*_o_^2^) + (0.040*P*)^2^] where *P* = (*F*_o_^2^ + 2*F*_c_^2^)/3
6662 reflections	(Δ/σ)_max_ = 0.001
419 parameters	Δρ_max_ = 0.42 e Å^−^^3^
0 restraints	Δρ_min_ = −0.46 e Å^−^^3^

### Special details

**Table:**

Geometry. All e.s.d.'s (except the e.s.d. in the dihedral angle between two l.s. planes) are estimated using the full covariance matrix. The cell e.s.d.'s are taken into account individually in the estimation of e.s.d.'s in distances, angles and torsion angles; correlations between e.s.d.'s in cell parameters are only used when they are defined by crystal symmetry. An approximate (isotropic) treatment of cell e.s.d.'s is used for estimating e.s.d.'s involving l.s. planes.
Refinement. Refinement of *F*^2^ against ALL reflections. The weighted *R*-factor *wR* and goodness of fit *S* are based on *F*^2^, conventional *R*-factors *R* are based on *F*, with *F* set to zero for negative *F*^2^. The threshold expression of *F*^2^ > σ(*F*^2^) is used only for calculating *R*-factors(gt) *etc*. and is not relevant to the choice of reflections for refinement. *R*-factors based on *F*^2^ are statistically about twice as large as those based on *F*, and *R*- factors based on ALL data will be even larger.

### Fractional atomic coordinates and isotropic or equivalent isotropic displacement parameters (Å^2^)

**Table:**

	*x*	*y*	*z*	*U*_iso_*/*U*_eq_	
Cu1	0.75479 (5)	0.39534 (4)	0.21433 (3)	0.04088 (17)	
Cu2	0.66890 (5)	0.61865 (4)	0.28275 (3)	0.04079 (17)	
O1	0.8527 (3)	0.3387 (3)	0.30458 (19)	0.0606 (9)	
O2	0.7892 (3)	0.5310 (3)	0.35870 (18)	0.0555 (8)	
O3	0.9072 (3)	0.4732 (2)	0.13965 (18)	0.0528 (8)	
O4	0.8294 (3)	0.6656 (2)	0.19611 (18)	0.0522 (8)	
O5	0.5672 (3)	0.6635 (3)	0.19240 (18)	0.0528 (8)	
O6	0.6483 (3)	0.4740 (3)	0.13148 (18)	0.0542 (8)	
O7	0.5860 (3)	0.3590 (2)	0.30257 (19)	0.0568 (8)	
O8	0.5185 (3)	0.5471 (3)	0.36340 (18)	0.0549 (8)	
N1	0.8234 (3)	0.2056 (3)	0.1613 (2)	0.0345 (8)	
N2	0.9035 (3)	−0.0403 (3)	0.1022 (2)	0.0394 (8)	
N3	0.5837 (3)	0.8058 (3)	0.3410 (2)	0.0356 (8)	
N4	0.4639 (3)	1.0502 (3)	0.4028 (2)	0.0399 (8)	
C1	0.6179 (4)	−0.0523 (4)	−0.1111 (3)	0.0513 (12)	
H1	0.5771	0.0294	−0.0902	0.062*	
C2	0.5709 (5)	−0.0916 (5)	−0.1708 (3)	0.0646 (13)	
H2	0.4991	−0.0361	−0.1900	0.078*	
C3	0.6274 (6)	−0.2106 (6)	−0.2026 (3)	0.0778 (16)	
H3	0.5931	−0.2376	−0.2419	0.093*	
C4	0.7352 (6)	−0.2896 (5)	−0.1760 (4)	0.0830 (18)	
H4	0.7771	−0.3698	−0.1992	0.100*	
C5	0.7832 (5)	−0.2519 (4)	−0.1149 (3)	0.0672 (14)	
H5	0.8553	−0.3076	−0.0962	0.081*	
C6	0.7237 (4)	−0.1307 (4)	−0.0814 (3)	0.0440 (10)	
C7	0.7775 (4)	−0.0940 (4)	−0.0172 (3)	0.0453 (11)	
H7	0.8378	−0.1597	0.0062	0.054*	
C8	0.7499 (4)	0.0227 (3)	0.0114 (2)	0.0403 (10)	
H8	0.6879	0.0893	−0.0100	0.048*	
C9	0.8096 (4)	0.0531 (3)	0.0735 (2)	0.0343 (9)	
C10	0.9545 (4)	−0.0093 (4)	0.1601 (3)	0.0427 (10)	
H10	1.0191	−0.0725	0.1815	0.051*	
C11	0.9155 (4)	0.1129 (4)	0.1895 (3)	0.0402 (10)	
H11	0.9545	0.1304	0.2299	0.048*	
C12	0.7714 (4)	0.1761 (3)	0.1035 (2)	0.0390 (10)	
H12	0.7071	0.2400	0.0822	0.047*	
C13	0.4607 (4)	0.8311 (4)	0.4013 (3)	0.0415 (10)	
H13	0.4144	0.7650	0.4233	0.050*	
C14	0.3986 (4)	0.9537 (4)	0.4329 (2)	0.0363 (9)	
C15	0.5867 (4)	1.0225 (4)	0.3440 (3)	0.0414 (10)	
H15	0.6346	1.0876	0.3228	0.050*	
C16	0.6474 (4)	0.9020 (4)	0.3128 (2)	0.0398 (10)	
H16	0.7343	0.8879	0.2713	0.048*	
C17	0.2624 (4)	0.9800 (4)	0.4969 (2)	0.0435 (11)	
H17	0.2174	0.9126	0.5163	0.052*	
C18	0.1986 (4)	1.0933 (4)	0.5294 (2)	0.0401 (10)	
H18	0.2462	1.1588	0.5097	0.048*	
C19	0.0615 (4)	1.1276 (4)	0.5929 (3)	0.0428 (10)	
C20	−0.0289 (4)	1.0469 (5)	0.6190 (3)	0.0556 (12)	
H20	−0.0013	0.9653	0.5975	0.067*	
C21	−0.1587 (5)	1.0852 (6)	0.6760 (3)	0.0763 (16)	
H21	−0.2177	1.0292	0.6930	0.092*	
C22	−0.2021 (6)	1.2048 (7)	0.7080 (3)	0.0865 (19)	
H22	−0.2912	1.2310	0.7453	0.104*	
C23	−0.1136 (6)	1.2863 (5)	0.6848 (4)	0.0830 (18)	
H23	−0.1418	1.3670	0.7077	0.100*	
C24	0.0172 (5)	1.2479 (4)	0.6276 (3)	0.0586 (13)	
H24	0.0767	1.3034	0.6119	0.070*	
C25	0.8544 (5)	0.4148 (4)	0.3563 (3)	0.0509 (12)	
C26	0.9394 (6)	0.3622 (4)	0.4186 (3)	0.0818 (17)	
H26A	0.9564	0.4326	0.4404	0.123*	
H26B	1.0257	0.3081	0.3879	0.123*	
H26C	0.8908	0.3121	0.4677	0.123*	
C27	0.9178 (4)	0.5848 (4)	0.1444 (3)	0.0418 (10)	
C28	1.0436 (4)	0.6262 (4)	0.0839 (3)	0.0619 (13)	
H28A	1.1215	0.5844	0.1053	0.093*	
H28B	1.0289	0.7185	0.0827	0.093*	
H28C	1.0608	0.6023	0.0250	0.093*	
C29	0.5753 (4)	0.5882 (4)	0.1377 (3)	0.0453 (11)	
C30	0.4905 (5)	0.6373 (4)	0.0744 (3)	0.0737 (15)	
H30A	0.4235	0.7153	0.0943	0.111*	
H30B	0.4440	0.5729	0.0723	0.111*	
H30C	0.5496	0.6551	0.0162	0.111*	
C31	0.5038 (4)	0.4367 (4)	0.3580 (3)	0.0457 (11)	
C32	0.3805 (5)	0.3961 (4)	0.4214 (3)	0.0717 (16)	
H32A	0.3654	0.4228	0.4793	0.108*	
H32B	0.3956	0.3036	0.4247	0.108*	
H32C	0.3010	0.4359	0.4012	0.108*	

### Atomic displacement parameters (Å^2^)

**Table:**

	*U*^11^	*U*^22^	*U*^33^	*U*^12^	*U*^13^	*U*^23^
Cu1	0.0487 (4)	0.0295 (3)	0.0416 (3)	−0.0043 (2)	−0.0094 (3)	−0.0116 (2)
Cu2	0.0456 (4)	0.0307 (3)	0.0416 (3)	−0.0063 (2)	−0.0045 (3)	−0.0122 (2)
O1	0.087 (2)	0.0403 (17)	0.058 (2)	−0.0011 (16)	−0.0324 (19)	−0.0128 (14)
O2	0.076 (2)	0.0442 (17)	0.0532 (19)	−0.0086 (16)	−0.0294 (18)	−0.0118 (14)
O3	0.0512 (19)	0.0387 (16)	0.0585 (19)	−0.0103 (14)	0.0030 (16)	−0.0153 (14)
O4	0.0499 (19)	0.0374 (16)	0.0569 (19)	−0.0111 (14)	0.0055 (16)	−0.0112 (14)
O5	0.059 (2)	0.0422 (16)	0.0555 (19)	0.0028 (14)	−0.0215 (17)	−0.0150 (14)
O6	0.066 (2)	0.0397 (16)	0.0569 (19)	0.0022 (15)	−0.0255 (17)	−0.0144 (14)
O7	0.065 (2)	0.0413 (16)	0.0542 (18)	−0.0195 (15)	0.0069 (17)	−0.0151 (14)
O8	0.057 (2)	0.0413 (17)	0.0573 (19)	−0.0153 (15)	0.0051 (16)	−0.0166 (14)
N1	0.035 (2)	0.0307 (17)	0.0353 (19)	−0.0036 (15)	−0.0090 (17)	−0.0041 (14)
N2	0.040 (2)	0.0305 (17)	0.046 (2)	−0.0077 (16)	−0.0092 (18)	−0.0083 (15)
N3	0.032 (2)	0.0368 (18)	0.0346 (19)	−0.0068 (15)	−0.0047 (17)	−0.0071 (15)
N4	0.044 (2)	0.0348 (18)	0.043 (2)	−0.0095 (16)	−0.0126 (18)	−0.0083 (15)
C1	0.048 (3)	0.056 (3)	0.052 (3)	−0.008 (2)	−0.019 (3)	−0.012 (2)
C2	0.056 (3)	0.084 (4)	0.060 (3)	−0.009 (3)	−0.031 (3)	−0.011 (3)
C3	0.084 (4)	0.092 (4)	0.077 (4)	−0.023 (4)	−0.037 (4)	−0.031 (3)
C4	0.104 (5)	0.068 (4)	0.095 (4)	−0.012 (3)	−0.043 (4)	−0.041 (3)
C5	0.065 (3)	0.047 (3)	0.099 (4)	0.003 (2)	−0.041 (3)	−0.028 (3)
C6	0.041 (3)	0.049 (2)	0.044 (2)	−0.010 (2)	−0.012 (2)	−0.010 (2)
C7	0.039 (3)	0.042 (2)	0.057 (3)	−0.005 (2)	−0.016 (2)	−0.013 (2)
C8	0.040 (3)	0.035 (2)	0.046 (2)	−0.0035 (19)	−0.015 (2)	−0.0061 (18)
C9	0.032 (2)	0.034 (2)	0.034 (2)	−0.0104 (18)	−0.0022 (19)	−0.0044 (17)
C10	0.041 (3)	0.039 (2)	0.049 (3)	−0.005 (2)	−0.018 (2)	−0.002 (2)
C11	0.045 (3)	0.038 (2)	0.040 (2)	−0.007 (2)	−0.016 (2)	−0.0070 (18)
C12	0.040 (3)	0.034 (2)	0.041 (2)	−0.0027 (19)	−0.014 (2)	−0.0068 (18)
C13	0.041 (3)	0.037 (2)	0.049 (3)	−0.0156 (19)	−0.009 (2)	−0.0104 (19)
C14	0.037 (2)	0.037 (2)	0.040 (2)	−0.0072 (18)	−0.014 (2)	−0.0119 (18)
C15	0.049 (3)	0.036 (2)	0.042 (2)	−0.016 (2)	−0.010 (2)	−0.0052 (19)
C16	0.040 (3)	0.043 (2)	0.036 (2)	−0.013 (2)	−0.009 (2)	−0.0017 (19)
C17	0.041 (3)	0.043 (2)	0.049 (3)	−0.017 (2)	−0.006 (2)	−0.013 (2)
C18	0.040 (3)	0.041 (2)	0.042 (2)	−0.0088 (19)	−0.012 (2)	−0.0110 (19)
C19	0.042 (3)	0.046 (2)	0.038 (2)	0.000 (2)	−0.015 (2)	−0.0087 (19)
C20	0.044 (3)	0.080 (3)	0.043 (3)	−0.013 (3)	−0.009 (2)	−0.018 (2)
C21	0.051 (3)	0.130 (5)	0.053 (3)	−0.028 (3)	−0.012 (3)	−0.015 (3)
C22	0.050 (4)	0.140 (6)	0.052 (3)	0.021 (4)	−0.015 (3)	−0.032 (4)
C23	0.079 (4)	0.075 (4)	0.075 (4)	0.025 (3)	−0.020 (4)	−0.033 (3)
C24	0.061 (3)	0.048 (3)	0.055 (3)	0.008 (2)	−0.013 (3)	−0.013 (2)
C25	0.067 (3)	0.044 (3)	0.043 (3)	−0.012 (2)	−0.020 (3)	−0.004 (2)
C26	0.116 (5)	0.066 (3)	0.076 (4)	0.001 (3)	−0.058 (4)	−0.014 (3)
C27	0.040 (3)	0.037 (2)	0.040 (2)	−0.001 (2)	−0.007 (2)	−0.001 (2)
C28	0.056 (3)	0.043 (3)	0.069 (3)	−0.008 (2)	0.003 (3)	−0.007 (2)
C29	0.042 (3)	0.049 (3)	0.043 (3)	−0.005 (2)	−0.012 (2)	−0.007 (2)
C30	0.085 (4)	0.067 (3)	0.075 (3)	0.004 (3)	−0.044 (3)	−0.016 (3)
C31	0.050 (3)	0.042 (2)	0.039 (3)	−0.013 (2)	−0.002 (2)	−0.004 (2)
C32	0.068 (3)	0.055 (3)	0.075 (3)	−0.032 (3)	0.023 (3)	−0.017 (3)

### Geometric parameters (Å, °)

**Table:**

Cu1—O6	1.947 (3)	C9—C12	1.389 (5)
Cu1—O1	1.956 (3)	C10—C11	1.377 (5)
Cu1—O3	1.968 (3)	C10—H10	0.9300
Cu1—O7	1.970 (3)	C11—H11	0.9300
Cu1—N1	2.181 (3)	C12—H12	0.9300
Cu1—Cu2	2.6077 (10)	C13—C14	1.397 (5)
Cu2—O4	1.938 (3)	C13—H13	0.9300
Cu2—O8	1.946 (3)	C14—C17	1.452 (5)
Cu2—O2	1.981 (3)	C15—C16	1.374 (5)
Cu2—O5	1.983 (3)	C15—H15	0.9300
Cu2—N3	2.191 (3)	C16—H16	0.9300
O1—C25	1.257 (4)	C17—C18	1.319 (5)
O2—C25	1.252 (5)	C17—H17	0.9300
O3—C27	1.252 (4)	C18—C19	1.461 (5)
O4—C27	1.264 (4)	C18—H18	0.9300
O5—C29	1.249 (4)	C19—C20	1.382 (5)
O6—C29	1.260 (4)	C19—C24	1.390 (5)
O7—C31	1.258 (4)	C20—C21	1.370 (6)
O8—C31	1.257 (4)	C20—H20	0.9300
N1—C11	1.323 (4)	C21—C22	1.365 (7)
N1—C12	1.326 (4)	C21—H21	0.9300
N2—C10	1.326 (4)	C22—C23	1.375 (7)
N2—C9	1.341 (5)	C22—H22	0.9300
N3—C16	1.325 (4)	C23—C24	1.380 (6)
N3—C13	1.330 (4)	C23—H23	0.9300
N4—C15	1.318 (5)	C24—H24	0.9300
N4—C14	1.340 (4)	C25—C26	1.489 (6)
C1—C2	1.367 (5)	C26—H26A	0.9600
C1—C6	1.370 (5)	C26—H26B	0.9600
C1—H1	0.9300	C26—H26C	0.9600
C2—C3	1.360 (6)	C27—C28	1.497 (5)
C2—H2	0.9300	C28—H28A	0.9600
C3—C4	1.364 (7)	C28—H28B	0.9600
C3—H3	0.9300	C28—H28C	0.9600
C4—C5	1.384 (6)	C29—C30	1.497 (6)
C4—H4	0.9300	C30—H30A	0.9600
C5—C6	1.394 (5)	C30—H30B	0.9600
C5—H5	0.9300	C30—H30C	0.9600
C6—C7	1.463 (5)	C31—C32	1.490 (5)
C7—C8	1.329 (5)	C32—H32A	0.9600
C7—H7	0.9300	C32—H32B	0.9600
C8—C9	1.447 (5)	C32—H32C	0.9600
C8—H8	0.9300		
			
O6—Cu1—O1	172.31 (11)	N1—C11—H11	119.5
O6—Cu1—O3	89.91 (13)	C10—C11—H11	119.5
O1—Cu1—O3	89.18 (13)	N1—C12—C9	122.3 (4)
O6—Cu1—O7	89.49 (13)	N1—C12—H12	118.9
O1—Cu1—O7	89.51 (13)	C9—C12—H12	118.9
O3—Cu1—O7	165.70 (10)	N3—C13—C14	122.3 (3)
O6—Cu1—N1	94.38 (11)	N3—C13—H13	118.9
O1—Cu1—N1	93.30 (12)	C14—C13—H13	118.9
O3—Cu1—N1	99.42 (11)	N4—C14—C13	119.9 (4)
O7—Cu1—N1	94.87 (11)	N4—C14—C17	118.8 (3)
O6—Cu1—Cu2	86.56 (8)	C13—C14—C17	121.3 (4)
O1—Cu1—Cu2	85.75 (8)	N4—C15—C16	123.2 (4)
O3—Cu1—Cu2	82.20 (8)	N4—C15—H15	118.4
O7—Cu1—Cu2	83.51 (8)	C16—C15—H15	118.4
N1—Cu1—Cu2	178.12 (8)	N3—C16—C15	120.9 (4)
O4—Cu2—O8	171.82 (10)	N3—C16—H16	119.6
O4—Cu2—O2	88.02 (13)	C15—C16—H16	119.6
O8—Cu2—O2	90.25 (13)	C18—C17—C14	124.1 (4)
O4—Cu2—O5	90.46 (13)	C18—C17—H17	117.9
O8—Cu2—O5	89.18 (12)	C14—C17—H17	117.9
O2—Cu2—O5	165.24 (11)	C17—C18—C19	127.3 (4)
O4—Cu2—N3	96.07 (11)	C17—C18—H18	116.3
O8—Cu2—N3	92.10 (11)	C19—C18—H18	116.3
O2—Cu2—N3	99.50 (11)	C20—C19—C24	117.8 (4)
O5—Cu2—N3	95.27 (11)	C20—C19—C18	123.0 (4)
O4—Cu2—Cu1	86.66 (8)	C24—C19—C18	119.2 (4)
O8—Cu2—Cu1	85.19 (8)	C21—C20—C19	121.1 (5)
O2—Cu2—Cu1	82.81 (8)	C21—C20—H20	119.5
O5—Cu2—Cu1	82.44 (8)	C19—C20—H20	119.5
N3—Cu2—Cu1	176.47 (9)	C22—C21—C20	120.6 (5)
C25—O1—Cu1	122.3 (3)	C22—C21—H21	119.7
C25—O2—Cu2	124.7 (3)	C20—C21—H21	119.7
C27—O3—Cu1	125.2 (3)	C21—C22—C23	119.8 (5)
C27—O4—Cu2	121.1 (3)	C21—C22—H22	120.1
C29—O5—Cu2	124.3 (3)	C23—C22—H22	120.1
C29—O6—Cu1	121.0 (3)	C22—C23—C24	119.7 (5)
C31—O7—Cu1	123.8 (3)	C22—C23—H23	120.2
C31—O8—Cu2	123.1 (3)	C24—C23—H23	120.2
C11—N1—C12	117.3 (3)	C23—C24—C19	121.1 (5)
C11—N1—Cu1	120.3 (3)	C23—C24—H24	119.5
C12—N1—Cu1	122.4 (3)	C19—C24—H24	119.5
C10—N2—C9	117.0 (3)	O2—C25—O1	124.2 (4)
C16—N3—C13	116.9 (3)	O2—C25—C26	118.4 (4)
C16—N3—Cu2	121.5 (3)	O1—C25—C26	117.4 (4)
C13—N3—Cu2	121.4 (2)	C25—C26—H26A	109.5
C15—N4—C14	116.8 (3)	C25—C26—H26B	109.5
C2—C1—C6	121.5 (4)	H26A—C26—H26B	109.5
C2—C1—H1	119.3	C25—C26—H26C	109.5
C6—C1—H1	119.3	H26A—C26—H26C	109.5
C3—C2—C1	121.0 (5)	H26B—C26—H26C	109.5
C3—C2—H2	119.5	O3—C27—O4	124.7 (4)
C1—C2—H2	119.5	O3—C27—C28	117.7 (3)
C2—C3—C4	118.9 (5)	O4—C27—C28	117.6 (4)
C2—C3—H3	120.5	C27—C28—H28A	109.5
C4—C3—H3	120.5	C27—C28—H28B	109.5
C3—C4—C5	120.8 (5)	H28A—C28—H28B	109.5
C3—C4—H4	119.6	C27—C28—H28C	109.5
C5—C4—H4	119.6	H28A—C28—H28C	109.5
C4—C5—C6	120.2 (5)	H28B—C28—H28C	109.5
C4—C5—H5	119.9	O5—C29—O6	125.6 (4)
C6—C5—H5	119.9	O5—C29—C30	117.4 (4)
C1—C6—C5	117.6 (4)	O6—C29—C30	117.1 (4)
C1—C6—C7	123.8 (4)	C29—C30—H30A	109.5
C5—C6—C7	118.6 (4)	C29—C30—H30B	109.5
C8—C7—C6	127.2 (4)	H30A—C30—H30B	109.5
C8—C7—H7	116.4	C29—C30—H30C	109.5
C6—C7—H7	116.4	H30A—C30—H30C	109.5
C7—C8—C9	124.4 (4)	H30B—C30—H30C	109.5
C7—C8—H8	117.8	O8—C31—O7	124.3 (4)
C9—C8—H8	117.8	O8—C31—C32	117.6 (4)
N2—C9—C12	120.1 (4)	O7—C31—C32	118.1 (4)
N2—C9—C8	118.7 (3)	C31—C32—H32A	109.5
C12—C9—C8	121.3 (4)	C31—C32—H32B	109.5
N2—C10—C11	122.5 (4)	H32A—C32—H32B	109.5
N2—C10—H10	118.8	C31—C32—H32C	109.5
C11—C10—H10	118.8	H32A—C32—H32C	109.5
N1—C11—C10	120.9 (4)	H32B—C32—H32C	109.5
			
O6—Cu1—Cu2—O4	−88.60 (13)	O8—Cu2—N3—C13	−6.8 (3)
O1—Cu1—Cu2—O4	91.53 (13)	O2—Cu2—N3—C13	−97.4 (3)
O3—Cu1—Cu2—O4	1.78 (12)	O5—Cu2—N3—C13	82.6 (3)
O7—Cu1—Cu2—O4	−178.48 (13)	C6—C1—C2—C3	0.3 (7)
O6—Cu1—Cu2—O8	92.11 (13)	C1—C2—C3—C4	−1.9 (8)
O1—Cu1—Cu2—O8	−87.75 (13)	C2—C3—C4—C5	2.5 (9)
O3—Cu1—Cu2—O8	−177.51 (13)	C3—C4—C5—C6	−1.6 (8)
O7—Cu1—Cu2—O8	2.24 (13)	C2—C1—C6—C5	0.7 (6)
O6—Cu1—Cu2—O2	−177.02 (13)	C2—C1—C6—C7	−179.6 (4)
O1—Cu1—Cu2—O2	3.11 (12)	C4—C5—C6—C1	0.0 (7)
O3—Cu1—Cu2—O2	−86.65 (13)	C4—C5—C6—C7	−179.7 (4)
O7—Cu1—Cu2—O2	93.10 (13)	C1—C6—C7—C8	−11.0 (7)
O6—Cu1—Cu2—O5	2.31 (12)	C5—C6—C7—C8	168.7 (4)
O1—Cu1—Cu2—O5	−177.56 (13)	C6—C7—C8—C9	−178.4 (3)
O3—Cu1—Cu2—O5	92.69 (13)	C10—N2—C9—C12	0.9 (5)
O7—Cu1—Cu2—O5	−87.57 (13)	C10—N2—C9—C8	−179.1 (3)
O3—Cu1—O1—C25	77.7 (3)	C7—C8—C9—N2	2.8 (6)
O7—Cu1—O1—C25	−88.1 (3)	C7—C8—C9—C12	−177.2 (4)
N1—Cu1—O1—C25	177.1 (3)	C9—N2—C10—C11	−0.7 (5)
Cu2—Cu1—O1—C25	−4.6 (3)	C12—N1—C11—C10	−0.4 (5)
O4—Cu2—O2—C25	−89.9 (4)	Cu1—N1—C11—C10	176.7 (3)
O8—Cu2—O2—C25	82.1 (4)	N2—C10—C11—N1	0.4 (6)
O5—Cu2—O2—C25	−5.6 (7)	C11—N1—C12—C9	0.6 (5)
N3—Cu2—O2—C25	174.3 (3)	Cu1—N1—C12—C9	−176.3 (3)
Cu1—Cu2—O2—C25	−3.0 (3)	N2—C9—C12—N1	−0.9 (6)
O6—Cu1—O3—C27	86.2 (3)	C8—C9—C12—N1	179.0 (3)
O1—Cu1—O3—C27	−86.2 (3)	C16—N3—C13—C14	1.4 (5)
O7—Cu1—O3—C27	−1.4 (7)	Cu2—N3—C13—C14	−173.6 (3)
N1—Cu1—O3—C27	−179.4 (3)	C15—N4—C14—C13	−0.5 (5)
Cu2—Cu1—O3—C27	−0.4 (3)	C15—N4—C14—C17	−179.4 (3)
O2—Cu2—O4—C27	79.1 (3)	N3—C13—C14—N4	−0.6 (6)
O5—Cu2—O4—C27	−86.2 (3)	N3—C13—C14—C17	178.2 (4)
N3—Cu2—O4—C27	178.5 (3)	C14—N4—C15—C16	0.8 (6)
Cu1—Cu2—O4—C27	−3.8 (3)	C13—N3—C16—C15	−1.0 (5)
O4—Cu2—O5—C29	84.6 (3)	Cu2—N3—C16—C15	173.9 (3)
O8—Cu2—O5—C29	−87.2 (3)	N4—C15—C16—N3	−0.1 (6)
O2—Cu2—O5—C29	0.6 (7)	N4—C14—C17—C18	−1.4 (6)
N3—Cu2—O5—C29	−179.3 (3)	C13—C14—C17—C18	179.8 (4)
Cu1—Cu2—O5—C29	−2.0 (3)	C14—C17—C18—C19	179.1 (4)
O3—Cu1—O6—C29	−85.7 (3)	C17—C18—C19—C20	−9.0 (7)
O7—Cu1—O6—C29	80.0 (3)	C17—C18—C19—C24	172.6 (4)
N1—Cu1—O6—C29	174.9 (3)	C24—C19—C20—C21	1.1 (7)
Cu2—Cu1—O6—C29	−3.5 (3)	C18—C19—C20—C21	−177.3 (4)
O6—Cu1—O7—C31	−88.9 (3)	C19—C20—C21—C22	0.4 (7)
O1—Cu1—O7—C31	83.5 (3)	C20—C21—C22—C23	−1.8 (8)
O3—Cu1—O7—C31	−1.3 (7)	C21—C22—C23—C24	1.7 (9)
N1—Cu1—O7—C31	176.8 (3)	C22—C23—C24—C19	−0.2 (8)
Cu2—Cu1—O7—C31	−2.3 (3)	C20—C19—C24—C23	−1.2 (7)
O2—Cu2—O8—C31	−85.9 (3)	C18—C19—C24—C23	177.3 (4)
O5—Cu2—O8—C31	79.4 (3)	Cu2—O2—C25—O1	0.5 (7)
N3—Cu2—O8—C31	174.6 (3)	Cu2—O2—C25—C26	−179.2 (3)
Cu1—Cu2—O8—C31	−3.1 (3)	Cu1—O1—C25—O2	3.8 (6)
O6—Cu1—N1—C11	172.9 (3)	Cu1—O1—C25—C26	−176.5 (3)
O1—Cu1—N1—C11	−7.4 (3)	Cu1—O3—C27—O4	−2.5 (6)
O3—Cu1—N1—C11	82.3 (3)	Cu1—O3—C27—C28	178.1 (3)
O7—Cu1—N1—C11	−97.2 (3)	Cu2—O4—C27—O3	4.8 (6)
O6—Cu1—N1—C12	−10.2 (3)	Cu2—O4—C27—C28	−175.8 (3)
O1—Cu1—N1—C12	169.5 (3)	Cu2—O5—C29—O6	−0.1 (6)
O3—Cu1—N1—C12	−100.8 (3)	Cu2—O5—C29—C30	179.5 (3)
O7—Cu1—N1—C12	79.7 (3)	Cu1—O6—C29—O5	3.2 (6)
O4—Cu2—N3—C16	−1.1 (3)	Cu1—O6—C29—C30	−176.4 (3)
O8—Cu2—N3—C16	178.5 (3)	Cu2—O8—C31—O7	2.4 (6)
O2—Cu2—N3—C16	87.9 (3)	Cu2—O8—C31—C32	−177.3 (3)
O5—Cu2—N3—C16	−92.2 (3)	Cu1—O7—C31—O8	0.7 (6)
O4—Cu2—N3—C13	173.6 (3)	Cu1—O7—C31—C32	−179.6 (3)
